# Supplementary material for: Saccharomyces boulardii (CNCM I-745) improves intestinal damage in sepsis by remodeling intestinal flora structure
Source: Front Cell Infect Microbiol. 2025 Jul 31;15:1602792. doi: 10.3389/fcimb.2025.1602792 (PMC12350329; doi:10.3389/fcimb.2025.1602792)
Supplement: Supplementary file 1 [file DataSheet1.zip › summary/summary/2_QC/CW0_4/CW0_4_len_dist.pdf]

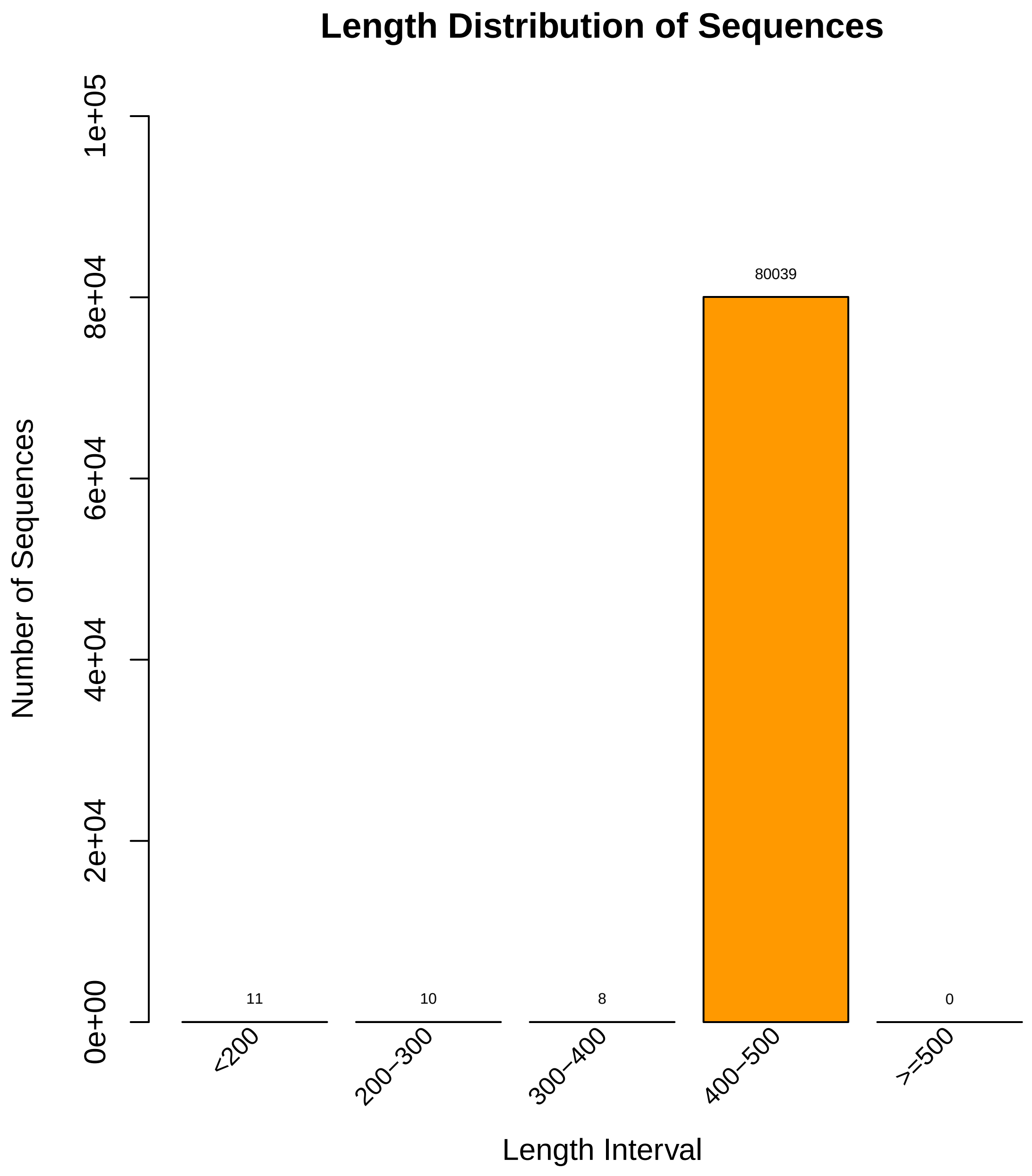

Length Distribution of Sequences
Number of Sequences
1e+05
8e+04
6e+04
4e+04
2e+04
0e+00
11
10
8
80039
0
<200
200–300
300–400
400–500
>=500
Length Interval
